# Alcohol use as a modifiable risk factor in cardiology: A qualitative study of patient perspectives in Sweden

**DOI:** 10.1371/journal.pone.0328990

**Published:** 2025-08-04

**Authors:** Paul Welfordsson, Anna-Karin Danielsson, Caroline Björck, Bartosz Grzymala-Lubanski, Kristina Hambraeus, Ida Haugen Löfman, Frieder Braunschweig, Matthias Lidin, Sara Wallhed Finn

**Affiliations:** 1 Department of Global Public Health, Karolinska Institutet, Solna, Sweden; 2 Department of Women’s and Children’s Health, Akademiska sjukhuset, Uppsala University, Uppsala, Sweden; 3 Centre for Research and Development, Region Gävleborg, Gävle, Sweden; 4 Department of Public Health and Clinical Medicine, Umeå University, Umeå, Sweden; 5 Department of Cardiology, Falun Hospital, Falun, Dalarna, Sweden; 6 Department of Medicine, Unit of Cardiology, Karolinska Institutet, Stockholm, Sweden; 7 Department of Cardiology, Heart and Vascular Center, Karolinska University Hospital, Stockholm, Sweden; 8 Unit of Clinical Alcohol Research, Institute of Clinical Research, University of Southern Denmark, Odense, Denmark; 9 Centre for Dependency Disorders, Stockholm, Sweden; Tribhuvan University Institute of Medicine, NEPAL

## Abstract

**Background:**

Alcohol use is an important cardiovascular risk factor and a major contributor to morbidity and mortality. Successful implementation of alcohol interventions in cardiology depends on patient acceptability.

**Objective:**

To understand patient perspectives on the feasibility of implementing alcohol interventions in cardiology services.

**Methods:**

Multi-site qualitative study. We conducted semi-structured interviews with a heterogenous-purposive sample of 15 adult cardiology patients with hazardous alcohol use. Participants were recruited from three geographically diverse regions in Sweden (Dalarna, Gävleborg, Stockholm) and were varied in terms of sociodemographic characteristics, cardiovascular diagnosis, risk factor profile, and level of alcohol use. We applied the Capability, Opportunity, Motivation and Behaviour (COM-B) system during coding and conducted a reflexive thematic analysis.

**Results:**

We identified 56 feasibility factors: 15 related to capability, 10 to opportunity, and 31 to motivation. Four themes emerged: 1. Alcohol use as relevant to cardiology, where participants recognized cardiovascular risk factors, expressed motivation for change, and identified a need to address alcohol use sensitively; 2. Aligning interventions with expectations and goals, where participants linked acceptability of alcohol interventions to personal goals and social norms; 3. Morbidity and shifting priorities, where participants prioritized quality of life and respect for autonomy; 4. Addressing barriers to alcohol dependence treatments, where participants saw a need to improve access to care.

**Conclusions:**

Findings suggest that alcohol interventions are acceptable to cardiology patients with hazardous alcohol use. Implementation strategies that prioritize quality of life, respect autonomy, and align with individual expectations and goals may be among the most acceptable. We also identified an opportunity to improve access to treatments for alcohol dependence within multidisciplinary heart teams or hospital-based addiction care services.

## Introduction

Alcohol use is an important modifiable cardiovascular risk factor and a major contributor to morbidity and mortality [1]. Healthcare interactions represent an opportunity to identify hazardous alcohol use and to offer interventions. The World Health Organization (WHO) recommends screening and brief interventions (SBI) as a population-based strategy to address hazardous alcohol use [2]. Since 2021, European cardiology guidelines have recommended that patients should be screened for alcohol use in all medical evaluations [3], yet evidence suggests that SBI is implemented inconsistently in clinical practice [4,5].

Alcohol screening typically involves the use of self-reported assessment methods, such as the WHO Alcohol Use Disorders Identification Test (AUDIT) questionnaire [6]. However, interest is growing in the use of objective, biomarker-based screening methods such as phosphatidylethanol (PEth) [7]. While questionnaire or biomarker screening can identify candidates for interventions, successful implementation depends on patient acceptability [8,9].

Patients who screen positive for hazardous alcohol use should be offered brief interventions (BIs) [10,11], which typically consist of motivational interviewing and can be delivered by non-specialist clinicians in around 5–10 minutes [12,13]. In practice, however, SBI activities can vary considerably, and may include standardized questions about alcohol use during clinical history taking, or informal conversations about lifestyle habits while performing investigations [14]. Meta-analyses indicate that BIs have modest, positive effects in general hospital settings [15,16], yet there is a paucity of studies evaluating their feasibility in cardiology. In previous research, we identified clinician-related barriers and facilitators to SBI in cardiology [17], but patient perspectives have not been studied. Findings from general hospital and primary care settings indicate that patients consider clinicians’ communication skills, the clinical environment, and the timing of SBI to be important factors affecting acceptability, yet it is unclear whether these issues apply in a cardiology context [18]. There is also evidence that individuals with hazardous drinking may experience conversations about alcohol use as uncomfortable [19,20]. There is a need to understand patients’ views and preferences to improve the implementation of BIs [21,22].

Patients who have symptoms of alcohol dependence – such as impaired control of alcohol use or a compulsion to drink – warrant diagnostic evaluation, and may require treatment for alcohol dependence [12]. Such patients may be referred to alcohol services, yet evidence suggests that many referrals do not lead to treatment [23]. It is important to understand patients’ perspectives to improve addiction services in hospital settings and bridge this care gap [24].

Patients’ views on the acceptability of alcohol interventions in cardiology may be shaped by their perceptions of alcohol as a cardiovascular risk factor [20,25,26]. Such perceptions are shaped by personal experiences, sociocultural contexts and media sources – a phenomenon known as lay epidemiology [27,28]. Pleasure and perceived social advantages are widely reported motivations for drinking alcohol [29–31]; lay epidemiology theory suggests that drinkers balance these supposed benefits against any perceived negative consequences of alcohol, including risks to health, and was applied in this study.

The application of theory in behavioural change studies provides a structure for optimizing interventions and implementation strategies. The Theoretical Domains Framework (TDF) synthesizes existing behavioural change theories and was refined by Cane and colleagues in 2012, who mapped each of its 14 domains to one of three pillars in a complementary model – the Capability, Opportunity, Motivation and Behaviour (COM-B) system – to develop an integrative model for cross-disciplinary implementation research [32]. The COM-B system is a widely-used framework that defines three inter-related factors required for a given behaviour: 1) Capability – an individual’s ability to enact a specific behaviour; 2) Opportunity, or external factors that contribute to a behaviour, and; 3) Motivation – cognitive processes affecting engagement in that behaviour [33]. Together, the TDF and COM-B provide an empirical basis for investigating implementation challenges and informing the development of effective interventions.

The aim of this study was to apply the COM-B framework to understand the feasibility of implementing alcohol interventions in cardiology services from patients’ perspectives. We answered the following research questions:

1. How do cardiology patients with hazardous alcohol use describe the acceptability of SBI and alcohol dependence treatments?
2. What views and preferences do these patients express regarding possible interventions?

## Methods

### Study design and ethical considerations

This qualitative study conformed to the Standards for Reporting Qualitative Research (SRQR, S1 File) [34]. A study protocol is publicly available [35]. The study was approved by the Swedish Ethical Review Authority (2022-02059-01).

Participation involved discussing sensitive personal information, such as alcohol use and health, which may trigger distress. To mitigate these risks, participants were informed about interview topics prior to providing verbal consent, and a clinical psychologist (SWF) was available to support any participants who experienced distress. Participants received written information about the study and provided verbal consent by responding to standardized consent statements which were audio-recorded prior to commencing interviews. Consent procedures were approved by the Swedish Ethical Review Authority. Interviews were conducted in private environments, and interviewers were not involved in patient care. Study materials were available in Swedish and English.

### Participants and setting

We included a heterogenous-purposive sample of 15 cardiology patients at hospitals in three geographically diverse regions in Sweden: Stockholm (n = 4), Sweden’s capital city; Gävle (n = 9), a medium-sized town, and Falun (n = 2), a small town/rural area. **Eligibility criteria included:** Age ≥ 18 years; hazardous alcohol use, defined as AUDIT scores ≥6 in women or ≥8 in men [11]; consenting to an individual interview; no physical, cognitive or mental health problems preventing interview participation; Swedish or English speaker.

Most participants (n = 12) were recruited from inpatient cardiology wards, while two were recruited from ambulatory care and one from an outpatient clinic. Participants had AUDIT scores ranging from eight to 23 points; Five had AUDIT scores that indicated probable alcohol dependence (≥13 in women or ≥15 in men). The burden of cardiovascular disease (CVD) varied widely, with diagnoses ranging from atrial fibrillation to multi-cardiovascular morbidity and advanced heart failure. Similarly, participants’ risk factor profiles ranged from isolated hazardous drinking to clusters of unhealthy lifestyle habits (Table 1).

**Table 1:** Participant characteristics.

Name^a^	Gender	Age	Education	Cardiovascular diagnosis	AUDIT score^b^	Alcohol use category^c^	Screened by staff	Smoking status	Diet^d^	Physical activity^e^	BMI category^f^
Lena	Woman	78	Secondary school	Atrial fibrillation	8	Hazardous use	No	Never-smoker	Healthy diet	Physically active	Healthy weight
Susanne	Woman	67	University	Heart failure, myocardial infarction, valvular disease, previous cardiac arrest	8	Hazardous use	Yes	Current smoker	Healthy diet	Physically inactive	Healthy weight
Magnus	Man	70	University	Myocardial infarction, previous coronary bypass and aortic aneurysm surgery, valvular disease, atrial flutter	8	Hazardous use	Yes	Ex-smoker	Unhealthy diet	Physically inactive	Obese
Jan	Man	75	Secondary school	Angina, valvular disease	9	Hazardous use	No	Ex-smoker	Unhealthy diet	Physically active	Overweight
Maria	Woman	58	Secondary school	Paroxysmal atrial fibrillation, valvular disease	9	Hazardous use	Yes	Never-smoker	Healthy diet	Physically inactive	Overweight
Annika	Woman	67	University	Heart failure, atrial fibrillation	10	Hazardous use	Yes	Ex-smoker	Unhealthy diet	Physically inactive	Obese
Elisabeth	Woman	80	Primary school	Myocardial infarction	11	Hazardous use	Yes	Ex-smoker	Unhealthy diet	Physically inactive	Healthy weight
Bengt	Man	66	Primary school	Atrial fibrillation, previous coronary bypass surgery	13	Hazardous use	No	Ex-smoker	Unhealthy diet	Physically inactive	Obese
Hans	Man	80	Secondary school	Heart failure, myocardial infarction, valvular disease, arrhythmia	13	Hazardous use	Yes	Ex-smoker	Unhealthy diet	Physically active	Obese
Stefan	Man	70	Secondary school	Myocardial infarction	14	Hazardous use	Yes	Ex-smoker	Healthy diet	Physically active	Overweight
Thomas	Man	67	Primary school	Heart failure, angina, previous coronary bypass surgery, stroke	17	Probable dependence	Yes	Ex-smoker	Unhealthy diet	Physically inactive	Overweight
Johan	Man	58	Secondary school	Heart failure, arrhythmia, intracranial haemorrhage	17	Probable dependence	Yes	Never smoker	Healthy diet	Physically inactive	Obese
Carina	Woman	65	Secondary school	Atrial fibrillation	17	Probable dependence	No	Never smoker	Healthy diet	Physically inactive	Overweight
Per	Man	80	Secondary school	Heart failure, atrial fibrillation	18	Probable dependence	Yes	Ex-smoker	Unhealthy diet	Physically inactive	Healthy weight
Erik	Man	82	Primary school	Atrial fibrillation, cardiac thrombus	23	Probable dependence	Yes	Ex-smoker	Unhealthy diet	Physically inactive	Overweight

a) All names are pseudonyms

b) Alcohol Use Disorders Identification Test (AUDIT), a 10-item self-report instrument for alcohol use

c) Hazardous alcohol use was defined as AUDIT scores ≥6 for women or ≥8 for men. Probable dependence was defined as AUDIT scores ≥13 in women or ≥15 in men

d) Diet was assessed using two items from a food index from the National Board of Health and Welfare in Sweden: “How often do you eat: 1.) vegetables, 2.) fruit?”. Response categories (≤once/week = 0 points; a few times/week = 1 point; once daily = 2 points; ≥ twice daily = 3 points) generated an overall score for diet (maximum 6 points). Diet was subsequently coded as a binary variable: scores <4 = unhealthy diet, ≥ 4 = healthy diet.

e) Physical activity was assessed using the moderate-vigorous physical activity component of the International Physical Activity Questionnaire, and coded as a binary variable: 0–149 minutes = physically inactive; ≥ 150 minutes = physically active.

f) BMI was calculated using self-reported height and weight (kg/m^2^) and coded as: < 18.5 = underweight; 18.5–24.9 = healthy weight; 25–29.9 = overweight; ≥ 30 = obese.

### Researcher characteristics

The research team included: a PhD student who is a medical doctor from the UK and who was the primary analyst (PW); a clinical psychologist with a background in clinical addiction treatment and a PhD in alcohol research who was second analyst (SWF). Additional analytic input was provided by: a public health scientist and alcohol researcher with a PhD (AKD); a clinical psychologist and researcher with a PhD and experience of hospital-based healthcare (CB); consultant cardiologists with PhDs (BGL, KH, IHL, FB), and a specialist cardiology nurse with a PhD (ML).

## Procedure

### Recruitment

Potential participants were identified during a previous study that assessed lifestyle factors (alcohol use, smoking, diet, physical activity, BMI) among cardiology patients in Sweden [36]. Those with hazardous alcohol use were given the opportunity to participate in an individual interview and asked to provide contact details if interested: Of 1107 survey participants, 86 (7.8%) had hazardous alcohol use, 60 were asked to express interest in interview participation, 20 provided contact details, and 15 completed an interview.

PW contacted potential participants to assess their eligibility and to provide verbal and written information about the study. Participants were offered two cinema tickets for interview completion. Interviews were conducted between 1^st^ December 2022–31^st^ August 2023.

### Data collection

Individual, semi-structured interviews were conducted in Swedish, either face-to-face at the hospital, or by telephone (by PW and SWF). Interviews followed an *a priori* topic guide (S2 File interview guide) and took around 30 minutes. We aimed to use lay terminology that was readily understandable by patients. Certain necessary jargon, such as ‘cardiovascular risk factor’, was explained to participants, however we did not differentiate between the concepts of screening, BIs, and treatment during conversations with patients. Rather, these concepts were inferred from participants’ responses. We defined screening as questions or other methods used to identify alcohol use and applied a broad definition of BIs, namely all structured conversations between patients and clinicians or other time-limited methods used to motivate individuals to reduce alcohol use. Alcohol treatment was defined as more intensive methods to address alcohol dependence, such as medication prescription or psychological treatments. Interviews were audio-recorded and transcribed verbatim.

### Stopping criteria

We conducted preliminary assessments of emerging data and applied the principles of information power [37]. Sample size was thus dependent on: 1) Variation in gender, age, socioeconomic position, AUDIT score and region; 2) The depth of interview discussions (‘data richness’); 3) The extent to which data could be mapped to a deductive codebook (described below); 4) The frequency with which emergent (inductive) codes were identified. In this context, information power reflects the adequacy of the sample to support meaningful theoretical insight, rather than relying on numeric sufficiency or thematic saturation alone. In practice, our sample of 15 participants was a compromise between the principles of information power and participant availability.

### Analysis

We conducted a reflexive thematic analysis [38]. We applied a deductive-inductive, three-phase, iterative approach to coding. First, PW processed all interview transcripts, assigning data systematically to top- and second-level codes using an *a priori* deductive codebook (S3 File *a priori* codebook) that consisted of COM-B ‘sources of behaviour’ (capability, opportunity, motivation; first-level codes) [33], mapped to the TDF (second-level codes) [32,39]. Simultaneously, we analyzed text inductively, applying lay epidemiology theory to identify emergent codes. In practice, this involved highlighting responses that were based on personal experiences, sociocultural contexts, and anecdotal reports, rather than formally-acquired knowledge. We aimed to assign all relevant interview material either to our deductive codebook or to emergent codes, which were agreed with a second analyst (SWF). Third-level codes (feasibility factors) were provisionally named, described, and added to the deductive coding matrix, while emergent codes were refined, expanded upon, and transferred to the deductive codebook where relevant. PW and SWF completed a joint round of coding to assess support for these codes within the empirical data. During this stage of analysis, we were able to map all codes – both deductive and inductive – to COM-B and the TDF, to compile a single, complete study codebook, which was agreed by consensus with the research team. Finally, third-level codes were analyzed inductively to identify shared meaning, grouped, and condensed into themes. These were named, described and reported as a narrative summary with selected quotations. We used Nvivo (release 1.7.1) to assist data analysis.

## Results

### Feasibility factors, according to COM-B

We identified a total of 56 feasibility factors, which we mapped to COM-B and the TDF. These included 15 codes related to capability, 10 to opportunity, and 31 to motivation. These feasibility factors are described in detail, with supporting quotations, in Table 2.

**Table 2:** Complete codebook.

Top level: COM-B	Second level: TDF	Third level: Feasibility factor	Description	Example quote
**Capability**				
	*Behaviour regulation*			
		Possible depressive symptoms	Reported symptoms which may be attributable to depression	“The thing is, if you feel no hope… then you might resort to various ‘cheats’, like a glass of wine with your pasta on Friday - It’s a bit nicer than not having a glass of wine. And then if it’s nice, maybe two glasses of wine. It becomes an escape if you are not feeling good.” (Johan)
		Symptoms of alcohol dependence	E.g., Cravings, compulsion to drink alcohol	“And then I was in the hospital here recovering, and – as you become healthier, there’s a devil sitting here whispering “now you can have a drink”.” (Erik)
		‘Sick quitter’	Reducing engagement in unhealthy lifestyle habit due to deteriorating health	“Since I became ill, I think about it more when I drink. Maybe I won’t take that extra half glass of wine, or that whiskey, but instead I’ll stay at a level that feels comfortable and cosy.” (Susanne)
		Becoming less able to take part in non-drinking activities	Loss of mobility, erosion of health and independence	“For the last ten years I have been going for walks with a friend. We would meet every morning at eight o’clock and then walk for an hour and a half. But I don’t know, since I turned 80 I started to feel so old – I wasn’t as strong. And then you feel irritated, when you’re not as fresh, so in the end I stopped going with him. And since then there has been more alcohol.” (Erik)
	*Beliefs about capabilities*			
		Beliefs about external help	Beliefs about need for intervention, or assertion that one must seek help for alcohol use oneself	“The doctors were very clear with me (about alcohol use) and I’m grateful for that, but it’s up to me in this case. Healthcare staff cannot give treatment... Well, they can make suggestions, but... yes” (Susanne)
		Expressing doubts about others’ self-reported alcohol use	Expressing doubts about the accuracy or honesty of how other people report alcohol use, while asserting that own self-reported alcohol use is accurate	”My guess is that most people either underreport their (alcohol) consumption, or deny it, because it is shameful to admit it. I’ve never kept it a secret – I’ve said it openly. “I have drunk alcohol. Yes, I drink alcohol.” (Hans)
		Difficulty estimating consumption	Perceived difficulty in accurately estimating alcohol consumption	“Based on a (PEth) blood test you can have a conversation about that it shows a high consumption of alcohol… Instead of answering a questionnaire, because that’s extremely difficult – there are these measures, and you have to count up the centiliters. It’s impossible because it’s all so different.” (Susanne)
		Too late to succeed in reducing alcohol use	Belief that too late in life or problems are too advanced to succeed in reducing alcohol use. Need to screen earlier in life.	“Say I’d been ten years or 20 years younger, then, hell, I would have tried how to quit because I would have 30 or 40 years left. But as a 67-year-old; Many people say, you fight to become smoke- and alcohol-free, it takes several years. And that is quite impossible. That’s why I think healthcare should take these tests, yes, when you’re younger and then continue now.” (Susanne)
	*Knowledge*			
		Awareness of own hazardous drinking	Recognising that own alcohol consumption meets threshold for hazardous drinking	“I understand that it (alcohol) is a risk factor and I am probably, I mean, I use alcohol a little too much for it to be good, I think.” (Annika)
		Identifying conversations about alcohol as interventions	Recollecting discussions about alcohol use with staff but not considering the conversation to be an intervention	“No, it has just been just the usual, when you sit at the doctor and talk about alcohol. Blah, blah, blah, and so on, and so on. But not anything in-depth, certainly not.” (Bengt)
		Information from healthcare sources	Reporting that information about risk factors acquired from clinical staff, heart school etc., rather than from web-based or media sources	“I have discussed these things with healthcare staff. But then some things, you have learned yourself then or read up on. And now I can’t tell the difference [laughs]... but it sticks somewhere in the head.” (Magnus) I haven’t searched on the web much, but I’ve met doctors for 15 years who have all said the same thing.” (Johan)
		Conceptualising alcohol as an important cardiovascular risk factor	Recognising that alcohol use is a cardiovascular risk factor – including for atrial fibrillation – while also attributing importance to other factors such as smoking and overweight	“It’s the usual old things: Bodyweight, what you eat and how much you move... Risk factors the top of the list, yeah that’s tobacco and alcohol. That’s how it is. But then, being overweight is also a very large risk factor” (Bengt)
		Accessing information from healthcare sources	conveyed by clinical staff, including in discussions on TV etc.	
	*Memory, attention and decision processes*			
		Prioritising other lifestyle factors	Reports of patients and staff prioritising other lifestyle habits over alcohol use. E.g., weight loss, exercise	“I think about being overweight on a daily basis [...] Seriously, I would really like some help with it.” (Annika) “My doctor at the time, whom I liked a lot – trusted – he thought I should go out and walk more… an hour every day, and so on.” (Elizabeth)
	*Skills*			
		Effective communication skills	Acknowledging the importance of clinical communication skills, including clear and direct questions about alcohol use	“She was good; She was matter-of-fact, without being pushy and all that. She was not bossy either – you sometimes get that from certain people. But she was good.” (Jan)
**Opportunity**				
	*Environmental context and resources*			
		Alcohol in the workplace	Consuming alcohol as part of work.	“I’m actually a sommelier so I’m trained in wine knowledge, drinking and tasting a lot of wine […] in connection with my profession.” (Jan)
		Limited time for SBI	Waiting times and perceived pressure on clinical services	“I had been there for seven hours, so I didn’t have the energy to talk (about alcohol).” (Thomas)
		Appreciating SBI during healthcare interactions	Discussions about alcohol use perceived as a positive aspect of interactions with healthcare staff	“It actually felt good that they had an eye on me. It’s worse if they don’t keep an eye on me, because after the brain haemorrhage I have a hard time keeping track of myself – I don’t have the best memory in the world.” (Johan)
		Retirement	Noting changes in alcohol use and healthcare opportunities to address these following retirement	“I’m not working anymore, so I drink more, but at the same time – I go to the hospital every week, so I don’t drink exactly every day.” (Thomas)
	*Social influences and support*			
		Being alone	Loneliness, boredom, or lack of support conceptualized as rational explanations or reasons for alcohol use	“I know it’s harmful for me to drink, but I have nothing to do all day. I just sit alone at home.” (Thomas)
		Family support	Acknowledging importance of support, but reporting difficulty discussing alcohol use with family	“I mostly talk to friends, actually […] not with my daughter. No [laughter], not with … no. But otherwise, we talk about most things.” (Elizabeth)
		Encountering alcohol in social contexts	Balancing positive dimensions of healthy social networks with expectation to drink alcohol. E.g., at dinners or parties	“I would think it would be really boring if I didn’t get to go to these dinners and party the way I do. I think that would reduce my quality of life enormously.” (Annika)
		Influence of partner	E.g., Importance of drinking when eating meals together, support from partner in moderating alcohol use	“We are trying to cut down to a normal level, just at weekends […] we have talked about it … together […] Yes, together with my wife. We push each other. Because it is probably more difficult if you sit alone, than if you are two. (Bengt)
		Support from healthcare staff	Including from primary healthcare and cardiology staff	I think I was at… just a health check with our GP. It was a great health centre at that time […] and the family had … We had such a good family doctor.
		Support groups	Preference for self-help alcohol/addiction support groups	“So, I was completely without (alcohol) when I left (treatment). That’s how it was. Then they had a support group and that you want to in your spare time. And these meetings – I did it, too.” (Carina)
**Motivation**				
	*Beliefs about consequences*			
		Alcohol and medication	Reports of cardiovascular medicines influencing drinking patterns. E.g., perceived or actual interactions with alcohol	“You can’t (drink) with this medicine. You have to go without alcohol for at least two weeks, to be able to take this medication for flickering (atrial fibrillation) […] Not one glass of wine… no alcohol at all, because then it can really get crazy, they said […] So they have talked to me about that. And you can’t lie about that – then it becomes dangerous.” (Carina)
		Importance of honest self-reporting of alcohol use	Noting that reporting alcohol use accurately is important for effective healthcare delivery or recovery	“I would say this: If you want to follow advice, you have to be honest. Why lie about things when you are trying to get treatment? It doesn’t work […] You have to be honest because then you can receive the right care.” (Stefan)
		Lay risk perception	Perception of risk in layman’s terms. E.g., single causes of CVD to be addressed; general non-specific malaise and loss of wellbeing associated with heavy drinking; ‘acceptable risk’ balanced against pleasure associated with alcohol consumption.	“You’re happiest when you don’t drink. And it’s more fun to wake up, too..” (Erik) “No one has said, “you exercise too little” or “your diet is not good enough” or … There is no one who has explicitly analyzed and had a conversation with me as well what is the reason why I got this calcification […] No one can say with hindsight that “the reason was this and therefore you should stop drinking alcohol or exercise more” or whatever it is. That analysis has not been done, and probably cannot be done either.” (Jan)
		Perceived medicinal effects of alcohol	Perceived benefits of alcohol use beyond pleasure or ‘quality of life’	“I think that … If I have a glass of wine or something, then I can fall asleep without (atrial) fibrillation. I think it has a calming effect […]. Perhaps if I’d had a sedative tablet it would have had the same effect. It’s helped me in a way, but still, I’ve gotten very high blood pressure instead. So that’s not good.” (Erik)
		Perceived priority or relative importance of risk factors	Beliefs, feelings and opinions about discussing alcohol use with clinical staff, in comparison to addressing other lifestyle habits	“I would say that I don’t have an alcohol problem, so I don’t really know if it’s relevant to discuss that... I just think that a line is crossed, that doctors and other alcohol educators think is appropriate [laughs]. I know that, but I don’t care […] I would like help with my excess bodyweight.” (Annika)
	*Emotions*			
		Acknowledging stressful life events and trauma	Including antecedent factors or perceived triggers providing ‘explanation’ for drinking	“I used to drink more… I had a partner who died four years ago […] Then I increased my alcohol consumption a bit. But now it has gotten better.” (Per)
		Difficulty acknowledging own drinking	Discomfort when cognizant of own hazardous drinking. Denial of alcohol use to self or others.	“Well, you lie to yourself, you lie to everyone. You lie about how much you drink or not. You’re being dishonest with yourself and you’re not being honest about … If I moved more, if I drank less, ate better food.” (Johan)
		Lacking energy required for lifestyle changes	Low energy due to physical or mental health problems, or old age	“Because I’m so tired, I can’t manage to move around much, but I’m standing and carrying around 125kg and… No, it’s heavy [..] I have a wife who tries to look after me… She tries her best, but it’s not easy with a stubborn old man...” (Bengt)
		Not caring about alcohol use	Expressing a lack of concern about own level of alcohol use	“I don’t care about it, because I also want to live while I’m still alive” (Annika)
		Reflections on aging, sickness and mortality	Advanced illness or age triggering thoughts to reconsider level of alcohol consumption. Altered risk perceptions in later life. E.g., Desire to have shorter, more enjoyable life or resignation to death.	“My god, I’m 80 years old. I really never thought I would be that old. My parents died when they were 60 – those were different times […] There is a certain resignation to it. But I’m still pretty careful otherwise… sort of.” (Elisabeth)
		Taking pride in openness about own alcohol use	Latent expressions of pride underlying reported openness about own alcohol use	“I’m not sensitive about that – even if other people can hear that it’s me, I’m going to be making anything up. If they hear who I am, so be it.” (Susanne)
	*Goals*			
		Respecting choice	Maintaining personal liberties and avoiding erosion of autonomy. Expressing wish to exercise control in life/death, including by drinking alcohol.	“I also think that one should respect, for example, ‘no, I can’t stop smoking”’ or ‘I choose to live this way”’. So you don’t get nagged about it. (Annika) “I don’t know how much time I have left to live. I think I can enjoy [laughs] what I do.“(Lena)
		Losing weight	Personal goal to lose weight	“Alcohol is a culprit regarding to the kilos, especially when it comes to the beers.” (Bengt)
		Moving towards discharge	Wanting to leave inpatient hospital environment as soon as possible. Focusing on treating acute illness rather than health promotion and prevention	“You just want to go home […] The less contact you have with others in the hospital, the better – you get to rest and sleep and read and go to the toilet. […] The staff, they do the best they can, and they’re happy if you don’t have too much to say, because they have 17 other patients to take care of… they do their job and they do it very well. But their job is not to hold my hand, their job is to care for someone who is sick.” (Johan)
		Preferring to manage alcohol use independently	Rather than seeking help or discussing with healthcare staff	“They said that if I had a drinking problem, there is help available at the (alcohol) dependence centre […] I didn’t want to go for it. I wanted to deal with it myself.” (Per)
		Quality of life	Perception that alcohol use increases quality of life. Motivation to drink for enjoyment or pleasure.	“Of course, wine, it has an intoxicating effect too. Plus, it’s a social… a way of keeping company, you hang out – it’s always like that with alcohol or wine […] I guess I like to have a little fun in life.” (Jan)
		Saving money	Economic motivation for lifestyle changes	“Yes, we can say that financially, it (drinking alcohol) is not good. Spirits are expensive.” (Stefan)
	*Intentions*			
		Harm reduction	Aiming to prevent or reduce harm, or to avoid short-term symptoms or reduce longer term risk	“It has gone too far. I have to change… my life. I’m just slowly getting worse and worse. And the easiest way to change is to remove the alcohol.” (Johan)
		Self-medication	Alcohol use for perceived relief of symptoms or other purported medicinal purposes	“Yes, I’ve had this anxiety […] a lot of (atrial) fibrillation. So then I bought a bottle of whiskey. I took two shots every night. But in the end there were three shots, and then four. I would have one or two shot maybe three weeks, and then I didn’t get so much fibrillation. And it was because the anxiety and stuff went away. They calmed down.” (Erik)
	*Optimism*			
		Overlooking lifestyle factors when well	Not considering CVD risk profile until after experiencing a cardiovascular event	“All these things you know about anyway... from TV, from radio, from books and everything. But you don’t notice until it’s out of control.” (Johan)
	*Reinforcement*			
		Effects of CVD on alcohol use	Including effects of diagnosis or symptoms on motivation for change. E.g., Changing lifestyle in conjunction with medical treatment, or reconsidering alcohol use in light of CVD diagnosis or symptoms.	“I’ve really been reconsidering things while I’ve been lying here these last few days… Maybe this was what I needed. Health and living, that’s the most important thing. That is the best motivation. You only have that. You only have one life, that’s how it feels. So now I’ve had a nudge in the right direction. That’s how it feels.” (Carina)
		Habits	Reporting repetitive patterns of behaviour that may be challenging to break.	“I’m a creature of habit. If you’ve been comfortable like that, you need to be a bit brave (to try to reduce alcohol use). But if you can get started with it, it’s probably good. And if you get good results then it kind of rolls on by itself in the end.” (Bengt)
		Personal, family or friends’ experiences	Influence of friends’ or own experiences on the acceptability of addressing alcohol use	“I had heard from a friend who got a tablet that took away the (alcohol) cravings. He thought of going to buy alcohol, but then when he stood in the queue, he thought to himself, “what the hell am I going to do here?” He wasn’t in the mood at all [laughs]. So he left. And then I thought, I’ll get hold of tablets like that. And now I’ve got them […] I took the first tablet yesterday.” (Erik)
		SBI perceived as a useful prompt	SBI conceptualized as a reminder to reconsider own alcohol use	“It feels good, and it also makes me aware. Even though I have been aware and know (about alcohol use as a risk factor) anyway, I think it’s good to be reminded […] I think it’s great that healthcare addresses alcohol and smoking.” (Susanne)
		Alcohol as a sensitive topic	Effects of societal norms, attitudes and values on the acceptability of SBI. Shame and stigma associated with addiction. Label avoidance	“If we are going to talk about personal things (alcohol use), then you shouldn’t sit in an open ward – in that case you should do it in a private room. […] The nurse or the doctor, well they’re bound by confidentiality, I guess.” (Stefan)
		Context is key	Attributing importance to the context in which drinking occurs. E.g., Normalizing drinking at weekends, with meals or in social contexts. Assigning relevance to the type of alcoholic beverage consumed. Assertion that celebration involves consuming alcohol.	“I almost never drink on weekdays, but I do drink at parties. And recently, when we have dinner, then I drink maybe once or twice a week… maybe a bottle of wine with dinner […] I only drink alcohol in positive contexts.” (Annika) “I drink a little alcohol sometimes. But only beer, no spirits… Yeah, I mostly drink beer at the weekends. I drink on weekdays sometimes too, but it’s not that often.” (Per)
		Taste as motivating factor	Reports of taste being an important motivation for alcohol consumption, reduced sense of taste in older age	“I don’t like all alcohol. I like a good red wine, decent red wine. That’s very important.” (Elisabeth)
		Unpleasant short-term symptoms after drinking	Focus on avoiding short-term rather than long-term complications of drinking: Hangovers, poor sleep quality, anxiety, palpitations etc.	“Of course (drinking alcohol) affects the heart in such a way that you can notice that you have drunk alcohol in a different way, so that you don’t feel so well maybe the next day and so on… I can feel my heart beating a little extra and so on […] sometimes in an unpleasant way.” (Annika)
	*Social and professional role and identity*			
		Exercising control over alcohol use	Expressing motivation to take responsibility or ownership for alcohol use	“I just decided I had to stop drinking so much. Because sometimes I drank so much that it was… sometimes I was even drunk, if I can say so.” (Stefan)
		Identity as a drinker	Identity defined in terms of alcohol use: E.g., as a drinker or a non-drinker, an ‘alcoholic’, or a party-person	“It’s all or nothing, that’s how it goes. That’s what I learned, too. That’s how it is for an alcoholic. You can’t believe you can have one drink. It’s all or nothing […] There is no turning back for me now.” (Carina)
		Too old for SBI	Reporting that it is too late in life to try to change lifestyle habits	“Time. Yes, I’ll say it again – why shouldn’t we be able to enjoy life when we are this old? We could be dead tomorrow.” (Lena)

### Themes

We identified four themes. These are summarised in Table 3 and are discussed in detail below.

**Table 3:** Theme summary and actionable recommendations.

Theme	Characteristics with regards to the feasibility of SBI	Actionable recommendations for policy and implementation
Alcohol use as relevant to cardiology	Links between alcohol use and CVDs. Trust in clinicians. Motivation for change following cardiovascular events. Addressing alcohol use sensitively.	Emphasize the acceptability of SBI during staff training. Empower cardiology nurses to deliver BIs in the outpatient clinic setting.
Aligning interventions with expectations and goals	Linking acceptability to personal goals. Gaining understanding through personal experiences. Awareness of own hazardous use. Adapting SBI to norms, attitudes, and values.	Tailor BIs to individual patient motivation and lifestyle goals. Embed SBI in multi-lifestyle interventions and cardiac rehabilitation programmes.
Morbidity and shifting priorities	Re-evaluating priorities in relation to aging, sickness, and mortality. Highlighting the importance of quality of life. Acknowledging challenges associated with increasing symptom burden and stressful life events. Respecting autonomy.	Utilize health-related and age-related opportunities for SBI, such as recent cardiac events or retirement from work.
Addressing barriers to alcohol dependence treatment	Challenges associated with self-reported measures of alcohol use. Beliefs about the need for external help. Taking ownership over alcohol use. Improving access to pharmacological treatments.	Consider biomarker-based screening methods, such as PEth. Develop and improve care pathways for alcohol dependence.

### Theme 1: Alcohol use as relevant to cardiology

In general, SBI was viewed as relevant to cardiology and as a helpful reminder to reflect on drinking habits. Participants felt comfortable discussing alcohol use with clinicians – in some cases more so than with family members. Support from clinicians was appreciated, and SBI was perceived as well-intentioned and helpful. Moreover, participants noted experiencing increased motivation to modify their drinking following cardiovascular events, in preparation for medical treatments, and after being diagnosed with conditions exacerbated by alcohol, such as atrial fibrillation.

“I’ve really been reconsidering things while I’ve been lying here these last few days… Maybe this was what I needed. Health and living, that’s the most important thing. That is the best motivation. You only have that. You only have one life, that’s how it feels. So now I’ve had a nudge in the right direction.” (Carina)

While SBI was reported to be acceptable by most participants, others expressed a degree of ambivalence, or resignation associated with experiencing CVD. In some cases, participants’ statements were suggestive of possible underlying depression.

“The thing is, if you feel no hope… then you might resort to various ‘cheats’, like a glass of wine with your pasta on Friday - It’s a bit nicer than not having a glass of wine. And then if it’s nice, maybe two glasses of wine. It becomes an escape if you are not feeling good.” (Johan)

Participants generally placed a high degree of trust in cardiology clinicians. Doctors were regarded as particularly well-placed to administer SBI and one participant suggested that doctors’ advice may carry additional weight. However, clinicians’ knowledge and skills were considered more important than their profession, and participants placed value on seeing the same clinician. There was also a strong tendency for participants to report acquiring knowledge about CVD risk factors from clinicians, rather than from online or media sources.

Participants identified opportunities for SBI during healthcare interactions and reported that SBI should be integrated within cardiology. Many expressed that screening should occur early in the care-flow process, although the importance of handling alcohol use sensitively was also strongly emphasized. Some participants expressed a fear of being stigmatized regarding their drinking, although – reassuringly – none of the participants reported discrimination or unfair treatment from healthcare staff. Nevertheless, participants indicated a need for privacy and suggested that the outpatient clinic may be a more appropriate environment for BIs than the open ward.

“If we are going to talk about personal things (alcohol use), then you shouldn’t sit in an open ward – in that case you should do it in a private room. […] The nurse or the doctor, well they’re bound by confidentiality, I guess.” (Stefan)

Participants emphasized the importance of effective communication and tended to prefer verbal interviews to questionnaire-based methods or digital screening tools. While one participant noted that questionnaires may provide a more complete picture of alcohol use, others reported difficulty estimating their alcohol consumption. Participants were also open to the use of biomarker screening methods – such as PEth blood tests – although once again, the importance of careful and sensitive implementation was emphasized. Biomarkers were perceived as providing a robust basis for interventions, and as a useful way to open the conversation around alcohol. Participants reflected that positive biomarker screening results may increase motivation for change, while negative screening results may offer reassurance. However, participants emphasized the need to respect autonomy and voiced concerns that biomarker screening may be perceived as an invasion of privacy. The need for transparency was emphasized, with informed consent being obtained before blood tests.

“Based on a (PEth) blood test you can have a conversation about that it shows a high consumption of alcohol… Instead of answering a questionnaire, because that’s extremely difficult – there are these measures, and you have to count up the centiliters. It’s impossible because it’s all so different.” (Susanne)

Participants viewed alcohol use as an important CVD risk factor and said that they expected to be asked about drinking, smoking and other lifestyle habits. Prescribing encounters were identified as opportunities for SBI; participants reported increased motivation to modify their drinking in light of possible interactions with new medications.

“You can’t (drink) with this medicine. You have to go without alcohol for at least two weeks […] Not one glass of wine […] because then it can really get crazy, they said […]. And you can’t lie about that – then it becomes dangerous.” (Carina)

### Theme 2: Aligning interventions with expectations and goals

Participants’ views and preferences highlighted the importance of aligning interventions with patients’ expectations and goals. Participants were generally aware of their own hazardous alcohol use, suggesting that generic interventions which focus on general health risks and drinking guidelines may fail to address individuals’ specific drivers of alcohol use. A range of motivations for alcohol use were cited, including pleasure, socialization, and celebration. Similarly, participants’ motivations for cutting down were not limited to health; two participants cited economic reasons. While participants were generally well-informed about potential negative health consequences of alcohol consumption, two participants reported drinking alcohol due to its perceived medicinal effects, including as an alternative to sleeping pills.

“If I have a glass of wine or something, then I can fall asleep without (atrial) fibrillation. I think it has a calming effect […]. Perhaps if I’d had a sedative tablet it would have had the same effect. It’s helped me in a way, but still, I’ve gotten very high blood pressure instead. So that’s not good.” (Erik)

Participants demonstrated knowledge of a range of CVD risk factors, although there appeared to be some variation in how the concept of risk was interpreted. Participants tended to focus on mitigating short-term risks when cutting down, such as avoiding hangovers and improving general wellbeing – rather than on modifying longer-term CVD risks. In addition to healthcare sources, participants’ knowledge of risk factors was often derived from personal experiences, or those of family and friends. These personal experiences tended to align with participants’ individual expectations and goals for CVD prevention, for example aiming to losing weight with the expectation of improving mobility or preventing diabetes.

“I could get diabetes, and you can also get CVD if you are severely overweight – I weighed 140kg. And that’s too much [...] My mother and father died from it.” (Carina)

Many participants identified interactions between lifestyle factors, such as the calorie-dense nature of alcoholic beverages or the tendency to drink less when exercising regularly. This suggests that there may be advantages to embedding SBI in multi-lifestyle CVD interventions that target patients’ self-reported goals.

“Alcohol is a culprit regarding to the kilos, especially when it comes to the beers.” (Bengt)

Participants reported overlooking their unhealthy lifestyle habits when well, and generally regarded other CVD risk factors, such as smoking, obesity, and physical inactivity, as being more important than alcohol use. However, all participants recognized that excessive alcohol consumption can be detrimental to health, and several participants reported feeling motivated to make lifestyle changes to minimize harm. While participants’ responses highlighted a need to consider individual motivations for alcohol use, responses also indicated a need to adapt SBI to societal norms, attitudes and values. For example, participants tended to normalize alcohol use in certain contexts, such as in social situations, with meals, or at weekends. In the same way, drinking wine or beer rather than spirits – particularly at levels that do not lead to visible intoxication – was generally viewed as acceptable.

“I almost never drink on weekdays, but I do drink at parties. And […] maybe a bottle of wine with dinner […]. I only drink alcohol in positive contexts.” (Annika)

### Theme 3: Morbidity and shifting priorities

Participants’ responses highlighted changes in alcohol use across the life course, and shifting priorities associated with multimorbidity in later life. Participants with advanced illness described two opposing directions in which age and morbidity may affect the acceptability of SBI. On the one hand, several participants described a deterioration in taste and a reduced tendency to drink alcohol – the so called ‘sick quitter’ effect. These participants described morbidity as a trigger to reconsider their drinking habits, and were generally open to SBI.

“Since I became ill, I think about it more when I drink. Maybe I won’t take that extra half glass of wine, or that whiskey, but instead I’ll stay at a level that feels comfortable and cosy.” (Susanne)

On the other hand, some participants reported lacking the energy required to successfully make lifestyle changes and feeling “too old” for SBI. These participants commented that SBI is more relevant during healthcare assessments for younger people.

“Say I’d been ten years or 20 years younger, then, hell, I would have tried how to quit because I would have 30 or 40 years left. But as a 67-year-old; Many people say, you fight to become smoke- and alcohol-free, it takes several years. And that is quite impossible. That’s why I think healthcare should take these tests, yes, when you’re younger and then continue now.” (Susanne)

Older and sicker participants tended to prioritize quality-of-life over harm reduction, and were generally less accepting of SBI. While most participants were aware that their level of alcohol use was associated with health risks, older and sicker participants tended to view these risks as less important than aspects such as the taste of alcoholic beverages or pleasure derived from drinking them.

“I don’t know how much time I have left to live. I think I can enjoy [laughs] what I do.“(Lena)

Nevertheless, many participants pointed out that their alcohol use had changed over time, often unintentionally. Retirement was noted as a common trigger for changes in alcohol use and may be an important opportunity for SBI; some participants reported increasing their alcohol use due to having more free time (“it’s Friday everyday”) and less responsibility to drive. Others reported a reduction in alcohol consumption after retirement, and pointed to the availability of alcohol and pressure to drink in certain jobs, such as those in the hospitality sector as risk factors for alcohol use. Changes in alcohol use were also reported in relation to disrupted social networks following retirement and isolation during the COVID-19 pandemic.

“I know it’s harmful for me to drink, but I have nothing to do all day. I just sit alone at home.” (Thomas)

Increasing symptom burden and stressful life events were reported as important drivers of alcohol use, and participants’ responses suggest that it may be important to acknowledge these during SBI. Participants reported experiencing physical and mental ill health that had resulted in profound losses of mobility and erosion of personal independence – limitations that prevented participation in non-drinking activities. Some participants who had experienced traumatic events tended to view these as antecedent factors, or triggers that provided a rational explanation for alcohol use. These participants tended to view SBI as less relevant to cardiology care.

“I used to drink more… I had a partner who died four years ago […] Then I increased my alcohol consumption a bit. But now it has gotten better.” (Per)

Overall, the importance of respecting autonomy and protecting choice was emphasized. Participants articulated their wishes to exercise control over their lives, in some cases by choosing to drink alcohol when other hedonic activities were no longer possible.

“I also think that one should respect, for example, ‘no, I can’t stop smoking”’ or ‘I choose to live this way”. So you don’t get nagged about it. (Annika)

### Theme 4: Addressing barriers to alcohol dependence treatments

Participants whose AUDIT scores indicated probable dependence described additional challenges with regards to alcohol use, such as cravings and a loss of control of drinking. These participants perceived barriers to alcohol dependence care, such as issues with self-reported screening methods, ambivalence regarding external help, and limited access to pharmacological treatments. Participants preferred direct questions about alcohol use, which were believed to facilitate accurate self-reporting. Participants stressed the importance of honest self-reporting, suggesting that this is an important step in exercising control over alcohol use. In some cases, openness about alcohol problems appeared to be source of pride for participants, perhaps reflecting a belief that they had ‘taken ownership’ of their drinking. Interestingly, several participants asserted that they always respond honestly to questions about alcohol use, yet suggested that others may not.

“My guess is that most people either underreport their (alcohol) consumption, or deny it, because it is shameful to admit it. I’ve never kept it a secret – I’ve said it openly. ‘I have drunk alcohol. Yes, I drink alcohol’.” (Hans)

Alcohol biomarkers were conceptualized as a useful tool which can help patients to overcome inaccurate or dishonest self-reporting of alcohol use – widely perceived as a barrier to treatment – by flagging the possibility of excessive alcohol consumption to clinicians. It was also suggested that cardiology patients may be more likely to disclose alcohol use to doctors than to other clinicians – possibly reflecting concerns that withholding this information may compromise the medical team’s ability to provide effective care.

“You have to be honest. Why lie about things when you are trying to get treatment? It doesn’t work […] You have to be honest because then you can receive the right care.” (Stefan)

Some participants expressed views that patients have a personal responsibility to seek help, and that those with alcohol dependence may struggle to acknowledge their own drinking. Nevertheless, responses generally suggested that SBI remains acceptable to those who believe that external help is not needed, and to those who prefer to manage their alcohol use independently.

“The doctors were very clear with me (about alcohol use) and I’m grateful for that, but it’s up to me in this case. Healthcare staff cannot give treatment... Well, they can make suggestions, but... yes” (Susanne)

Participants with probable alcohol dependence reported experiencing a range of alcohol dependence treatments, including support groups, psychological treatments, medications, and residential treatments. Overall, these participants reported a need for improved collaboration between cardiology services, addiction care, and primary care. In particular, participants highlighted a role for improved access to pharmacological treatments, and expressed a preference for this to be available at the cardiology department.

“I’d be happy if I got it (pharmacological treatment) at once […] even if it’s from a cardiologist. It would be easier to get it here, because… if, when I’m being discharged, they say, “Ok, now you can go to the addiction treatment centre”, then I will promise to go there. But then, before I get there, I might have different idea. So, it’s better to prescribe it here.” (Erik)

## Discussion

This qualitative study mapped cardiology patients’ views on SBI to the COM-B system. In general, SBI was acceptable, and participants identified implementation opportunities within routine care. Four themes emerged: alcohol use as relevant to cardiology; aligning interventions with expectations and goals; morbidity and shifting priorities, and addressing barriers to alcohol dependence treatment. Overall, participants highlighted the importance of effective communication and of individualizing preventive cardiology.

Our finding that cardiology patients with hazardous alcohol use view SBI as acceptable is consistent with studies in general hospitals [18,20,40], primary care [41–43], and in the general public in Scandinavia [44]. These results are further consistent with a broader picture of acceptability regarding lifestyle modification strategies [45], including smoking cessation [46] and dietary interventions, in cardiology settings [47]. Our study identified challenges surrounding self-reported measures of alcohol use, yet participants were generally accepting of questionnaire and biomarker screening methods – a finding also reported in primary care [48,49]. Like those studies, our findings highlight the importance of handling SBI sensitively. Earlier work suggests that some patients experience discomfort when discussing alcohol use [20], and that risky drinkers may have negative attitudes towards discussing alcohol with clinicians [19,50]. Groves et al reported that patients felt that doctors were dismissive of their alcohol problems, and preferred interactions with nurses [18]. In contrast, participants in our study described positive healthcare experiences, and considered doctors well-placed to administer SBI. This may suggest that alcohol stigma is reducing, or is context specific. Our findings balance clinicians’ concerns about being perceived as judgemental or of harming the clinician-patient relationship [17]. Still, participants emphasized the importance of clear communication and privacy, suggesting that the outpatient cardiology clinic may be an appropriate context for BIs. Nurse-led outpatient consultations may be a particularly promising environment for BIs, since cardiology nurses may already be skilled in behaviour change strategies such as motivational interviewing. Given that cardiologists are typically responsible for prescribing medications and other medical aspects of care, outpatient cardiology nurses may also have more time to dedicate to addressing lifestyle habits such as alcohol use.

Our findings highlight the importance of tailoring SBI to the individual, a finding also reported by Groves et al [18]. Participants recognised that alcohol use has negative health consequences, were generally aware of their own hazardous use, and described a range of motivations for reducing consumption. These motivations were typically related to participants’ lived experiences and included weight loss and saving money; incentives that were also reported in the Alcohol Toolkit Study [51]. Participants also drew on pervasive norms, attitudes, and values surrounding alcohol. For example, binge drinking at parties was viewed as less problematic than drinking alone at home – a phenomenon also reported in Australia [52] – despite conferring equivalent somatic health risks. Our findings suggest that, to ensure relevance to patients, BIs should be embedded in multi-lifestyle interventions that target patients’ self-reported goals, and should strike a balance between addressing the quantity of alcohol consumed, the context in which drinking takes place, and the consequences of alcohol use. Cardiac rehabilitation programmes may be an appropriate setting for such interventions, given their multi-modal nature.

We found that older and sicker participants, particularly those who believed they were approaching the final years of their life, were generally less accepting of SBI, and tended to prioritize pleasure, “quality of life”, and self-determination, over harm reduction. This finding has been reported previously [53,54] and is consistent with lay epidemiology theory, which suggests that subjective experiences are an influential contributor to risk perceptions and health behaviours [27]. Additionally, we found that some older patients believe that it is ‘too late’ to cut down, a finding that has been reported previously [55]. Importantly, this belief is not supported by empirical evidence; Studies report favourable outcomes with SBI among older adults [56], and the American Heart Association recommends multi-component behaviour change interventions for middle-aged and older adults [57]. Nevertheless, our findings emphasize that SBI should be tailored to the individual [43], and suggest that quality of life may be an important discussion point with cardiology patients.

Our study illustrates the breadth of needs among cardiology patients with hazardous alcohol use, and suggests that there is an opportunity to integrate alcohol dependence treatments into cardiology, for example in a multidisciplinary heart team or hospital-based addiction care service [24]. Such care may include pharmacotherapy, which is underutilized in clinical practice [58,59]. Participants’ experiences mirrored earlier work that casts doubt on the effectiveness of referring patients to addiction services, with many failing to receive treatment [60]. These findings are consistent with results from a qualitative study of cardiology clinicians in Sweden [17] and reinforce wider calls to strengthen addiction care in general hospital settings [24].

An emerging approach to SBI involves screening for alcohol use with PEth [49], – a direct biomarker of alcohol consumption – to obtain an objective indication of alcohol use over the previous 2–4 weeks [61]. A major advantage of PEth is that screening results are not susceptible to underreporting and recall bias. However, ethical and legal aspects must be considered carefully before widespread implementation of biomarker-based alcohol screening methods [62]. Possible patient concerns, such as disruption to the healthcare staff/patient relationship or feeling stigmatized also need to be taken into consideration.

Our findings are from a Swedish context and we note that the acceptability of SBI may vary internationally [50]. However, a degree of transferability is anticipated. For example, increasing symptom burden in older age is a common problem in countries with aging populations, and barriers to alcohol dependence treatments are reported widely, with the majority of those affected not receiving care [63,64].

### Strengths and limitations

Study strengths included recruitment of adults with hazardous alcohol use – a group that tends to be underrepresented in research. However, while we applied the principles of information power, we acknowledge that our sample of 15 interviews may not capture the full heterogeneity of patient perspectives across underrepresented groups or different cultural norms regarding alcohol use. A further limitation was that we did not manage to recruit younger participants, even though hazardous alcohol use is more prevalent among younger cardiology patients [36]. Our findings thus reflect the views of older adults and may not be consistent with those of younger patients or those who are not receiving hospital-based care. We also note that the views of participants may differ from those who declined to participate. This may have introduced bias into our results, since awareness of hazardous drinking and perceptions of stigma may have influenced participants’ views on SBI acceptability, as well as their decision to participate in a research interview. Interpretative limitations included our intentionally broad definitions of alcohol screening and BIs, which aimed to facilitate a wider dialogue around SBI but may have led to confusion among some participants. We also note that participants had hazardous alcohol use, and that non-drinkers’ views on SBI may differ. However, previous studies indicate that acceptability for SBI is high among general hospital patients [40,45] – our study adds evidence that this may also be the case among cardiology patients with hazardous alcohol use.

## Conclusions

This study suggests that SBI is acceptable to cardiology patients with hazardous alcohol use, who viewed alcohol use as relevant to cardiovascular care. However, we found that older cardiology patients and those with advanced disease may be less accepting of SBI. Our findings suggest that interventions and implementation strategies that prioritize quality of life, respect autonomy, and align with individual expectations and goals may be among the most acceptable to cardiology patients. We also identified an opportunity to improve access to treatments for alcohol dependence within multidisciplinary heart teams or hospital-based addiction care services. Future research should prioritize capturing the perspectives of younger individuals and those in the earlier stages of CVD to optimize secondary prevention strategies.

## Supporting information

S1 File Checklist. (DOCX)

S2 File Interview guide. (DOCX)

S3 File *A priori* codebook. (DOCX)
