# Supplementary material for: Alcohol use as a modifiable risk factor in cardiology: A qualitative study of patient perspectives in Sweden
Source: PLoS One. 2025 Aug 4;20(8):e0328990. doi: 10.1371/journal.pone.0328990 (PMC12321063; doi:10.1371/journal.pone.0328990)
Supplement: S2 File — (DOCX) [file pone.0328990.s002.docx]

**S2: Interview Guide**

1. Please tell me a little about yourself:
   - Age?
   - education/profession?
   - cardiovascular diagnosis/diagnoses?
   - Treatment and timeline?
2. What cardiovascular risk factors do you know?
   - Relative importance of risk factors?
   - Lifestyle habits and other risk factors?
3. How do you relate to these risk factors?
   - Do you have any cardiovascular risk factors?
   - Relative importance of these?
   - Perceived advantages and disadvantages of making changes to lifestyle habits?
   - Has anything affected the way you think and act with regard to lifestyle habits? Effects of cardiovascular diagnosis?
4. How did you become aware of these risk factors?
5. What are your views on preventive interventions for unhealthy lifestyle habits in cardiology wards and clinics?
   - Have you been asked about your lifestyle habits or offered advised on these by cardiology staff?
   - How did you find that experience? Is it acceptable? Is it relevant to cardiology?
   - How do you feel that preventive work for unhealthy lifestyles habits fits in with other treatments in the cardiology department?
6. What are your views on alcohol prevention in cardiology wards and clinics?
   - Have you been asked about drinking alcohol or offered advised on this by cardiology staff?
   - How did you find that experience? Is it acceptable? Is it relevant to cardiology?
   - How do you feel that alcohol prevention fits in with other treatments in the cardiology department?
7. How would you prefer to be asked about alcohol?
   - What aspects are important to consider?
   - What aspects are acceptable and relevance to address in cardiology?
   - What methods for assessing alcohol use do you know? (questionnaire/interview/biomarker/digital tools/other)
   - What advantages and disadvantages do you see with these methods?
   - Which staff should be responsible for addressing alcohol use in cardiology?
   - In what environment and at what point in your care?

8. Your survey responses indicate that you had hazardous alcohol use? Were you aware that your alcohol habits could present a risk to your health?

9. Is there anything else that you would like to add?
