# Supplementary material for: Alcohol use as a modifiable risk factor in cardiology: A qualitative study of patient perspectives in Sweden
Source: PLoS One. 2025 Aug 4;20(8):e0328990. doi: 10.1371/journal.pone.0328990 (PMC12321063; doi:10.1371/journal.pone.0328990)
Supplement: S3 File — (DOCX) [file pone.0328990.s003.docx]

**S3: *a priori* Deductive Codebook**

Top-level codes (COM-B headings, indicated below in bold) were mapped to second-level codes (the 14 domains of the TDF, in *italic*s) by Cane and colleagues (1) to create a deductive codebook that has been used previously by our research team (2). Descriptions were derived from definitions within the American Psychological Associations’ Dictionary of Psychology (3).

| Code | | |  |
| --- | --- | --- | --- |
| Top level:  COM-B | Second level: TDF | Third level: Individual feasibility factor | Description |
| **Capability** |  |  |  |
|  | *Behaviour regulation* |  | *Anything aimed at managing or changing objectively observed or measured actions. Component constructs: Self-monitoring Breaking habit Action planning* |
|  | *Beliefs about capabilities* |  | *Acceptance of the truth, reality, or validity about an ability, talent, or facility that a person can put to constructive use. Component constructs: Self-confidence Perceived competence Self-efficacy Perceived behavioural control Beliefs Self-esteem Empowerment Professional confidence* |
|  | *Knowledge* |  | *An awareness of the existence of something. Component constructs: Knowledge (including knowledge of condition /scientific rationale) Procedural knowledge Knowledge of task environment* |
|  | *Memory, attention and decision processes* |  | *The ability to retain information, focus selectively on aspects of the environment and choose between two or more alternatives. Component constructs: Memory Attention Attention control Decision making Cognitive overload / tiredness* |
|  | *Skills* |  | *An ability or proficiency acquired through practice. Component constructs: Skills Skills development Competence Ability Interpersonal skills Practice Skill assessment* |
| **Opportunity** |  |  |  |
|  | *Environmental context and resources* |  | *Any circumstance of a person's situation or environment that discourages or encourages the development of skills and abilities, independence, social competence, and adaptive behaviour. Component constructs: Environmental stressors Resources / material resources Organisational culture /climate Salient events / critical incidents Person x environment interaction Barriers and facilitators* |
|  | *Social influences and support* |  | *Those interpersonal processes that can cause individuals to change their thoughts, feelings, or behaviours. Components constructs: Social pressure Social norms Group conformity Social comparisons Group norms Social support Power Intergroup conflict Alienation Group identity Modelling* |
| **Motivation** |  |  |  |
|  | *Beliefs about consequences* |  | *Acceptance of the truth, reality, or validity about outcomes of a behaviour in a given situation. Component constructs: Beliefs Outcome expectancies Characteristics of outcome expectancies Anticipated regret Consequents* |
|  | *Emotions* |  | *A complex reaction pattern, involving experiential, behavioural, and physiological elements, by which the individual attempts to deal with a personally significant matter or event. Component constructs: Fear Anxiety Affect Stress Depression Positive / negative affect Burn-out* |
|  | *Goals* |  | *Mental representations of outcomes or end states that an individual wants to achieve. Component constructs: Goals (distal / proximal) Goal priority Goal / target setting Goals (autonomous / controlled) Action planning Implementation intention* |
|  | *Intentions* |  | *A conscious decision to perform a behaviour or a resolve to act in a certain way. Component constructs: Stability of intentions Stages of change model Transtheoretical model and stages of change* |
|  | *Optimism* |  | *The confidence that things will happen for the best or that desired goals will be attained). Component constructs: Optimism Pessimism Unrealistic optimism Identity* |
|  | *Reinforcement* |  | *Increasing the probability of a response by arranging a dependent relationship, or contingency, between the response and a given stimulus. Component constructs: Rewards (proximal / distal, valued / not valued, probable / improbable) Incentives Punishment Consequents Reinforcement Contingencies Sanctions* |
|  | *Social and professional role and identity* |  | *A coherent set of behaviours and displayed personal qualities of an individual in a social or work setting. Component constructs: Professional identity Professional role Social identity Identity Professional boundaries Professional confidence Group identity Leadership Organisational commitment* |

**References:**

1. Cane J, O’Connor D, Michie S. Validation of the theoretical domains framework for use in behaviour change and implementation research. Implementation Science. 2012;7(1):37.

2. Welfordsson P, Danielsson AK, Björck C, Grzymala-Lubanski B, Hambraeus K, Lidin M, et al. Feasibility of alcohol interventions in cardiology: A qualitative study of clinician perspectives in Sweden. Eur J Cardiovasc Nurs. 2024.

3. APA Dictionary of Psychology. VandenBos GR, editor. Washington, DC, US: American Psychological Association; 2007. xvi, 1024-xvi, p.
